# Role of Vitamin D in Maintaining Renal Epithelial Barrier Function in Uremic Conditions

**DOI:** 10.3390/ijms18122531

**Published:** 2017-11-26

**Authors:** Milos Mihajlovic, Michele Fedecostante, Miriam J. Oost, Sonja K. P. Steenhuis, Eef G. W. M. Lentjes, Inge Maitimu-Smeele, Manoe J. Janssen, Luuk B. Hilbrands, Rosalinde Masereeuw

**Affiliations:** 1 Division of Pharmacology, Utrecht Institute for Pharmaceutical Sciences, Utrecht University, 3584 CG Utrecht, The Netherlands; m.mihajlovic@uu.nl (M.M.); m.fedecostante@uu.nl (M.F.); m.j.oost@students.uu.nl (M.J.O.); s.k.p.steenhuis@students.uu.nl (S.K.P.S.); m.j.janssen1@uu.nl (M.J.J.); 2 Department of Clinical Chemistry and Haematology, University Medical Centre Utrecht, 3584 CX Utrecht, The Netherlands; egwm.lentjes@umcutrecht.nl (E.G.W.M.L.); imaitimu@umcutrecht.nl (I.M.-S.); 3 Department of Nephrology, Radboud University Medical Center, 6525 GA Nijmegen, The Netherlands; luuk.hilbrands@radboudumc.nl

**Keywords:** bioartificial kidney, conditionally immortalized proximal tubule cells, chronic kidney disease, end-stage renal disease, vitamin D, uremic toxins, epithelial barrier

## Abstract

As current kidney replacement therapies are not efficient enough for end-stage renal disease (ESRD) treatment, a bioartificial kidney (BAK) device, based on conditionally immortalized human proximal tubule epithelial cells (ciPTEC), could represent an attractive solution. The active transport activity of such a system was recently demonstrated. In addition, endocrine functions of the cells, such as vitamin D activation, are relevant. The organic anion transporter 1 (OAT-1) overexpressing ciPTEC line presented 1α-hydroxylase (CYP27B1), 24-hydroxylase (CYP24A1) and vitamin D receptor (VDR), responsible for vitamin D activation, degradation and function, respectively. The ability to produce and secrete 1α,25-dihydroxy-vitamin D_3_, was shown after incubation with the precursor, 25-hydroxy-vitamin D_3_. The beneficial effect of vitamin D on cell function and behavior in uremic conditions was studied in the presence of an anionic uremic toxins mixture. Vitamin D could restore cell viability, and inflammatory and oxidative status, as shown by cell metabolic activity, interleukin-6 (IL-6) levels and reactive oxygen species (ROS) production, respectively. Finally, vitamin D restored transepithelial barrier function, as evidenced by decreased inulin-FITC leakage in biofunctionalized hollow fiber membranes (HFM) carrying ciPTEC-OAT1. In conclusion, the protective effects of vitamin D in uremic conditions and proven ciPTEC-OAT1 endocrine function encourage the use of these cells for BAK application.

## 1. Introduction

It has been reported that chronic kidney disease (CKD), defined as the sustained presence of a decreased glomerular filtration rate (GFR) with or without increased albumin excretion, has a rather high global prevalence, estimated to be between 11% and 13% [1]. The progressive loss of kidney function will ultimately lead to a permanent state of end-stage renal disease (ESRD). Kidney failure is accompanied by a noticeable accumulation of a variety of endogenous uremic metabolites that are not efficiently cleared by the kidneys, leading to a broad range of pathologies, mostly cardiovascular disease and bone disorders, with reduced quality of life, as well as significantly increased mortality [2,3,4]. Although kidney transplantation is the treatment of choice for most patients with ESRD, patients who are older or have significant comorbidity are not eligible for transplantation. Moreover, due to a shortage of donor organs, dialysis therapy is frequently required during the waiting time for transplantation. However, both hemodialysis and peritoneal dialysis are inefficient techniques for the removal of waste products, especially larger and protein-bound uremic toxins [5]. Moreover, hemodialysis decreases the amount of vitamin D in serum [6].

The kidney also has intrinsic endocrine activity, producing hormones and immunomodulatory molecules. One of the essential hormones is 1α,25-dihydroxy-vitamin D_3_ (1,25(OH)_2_D_3_; calcitriol), the most active form of vitamin D, which is often deficient in CKD and ESRD populations, giving rise to severe comorbidities [7,8]. Normally, 25-hydroxy-vitamin D_3_ (25(OH)D_3_), mostly bound to vitamin D binding protein (VDBP), is taken up in proximal tubular epithelial cells (PTEC) by the multiligand binding receptor megalin (also known as low-density lipoprotein-related protein 2; LRP2) from the glomerular ultrafiltrate and subsequently converted by 1α-hydroxylase (CYP27B1) to 1,25(OH)_2_D_3_. 1,25(OH)_2_D_3_ has both autocrine and endocrine functions [8,9], and by binding to the intracellular vitamin D receptor (VDR) it is able to control the expression of genes involved in the regulation of skeletal health, but it can also have a range of other functions with effects on the cardiovascular and immune systems [10,11,12]. The activity of 24-hydroxylase (CYP24A1) is responsible for maintaining vitamin D homeostasis when present in high serum concentrations, as it catalyzes 1,25(OH)_2_D_3_ oxidation to inactive metabolites in PTEC that can be excreted in the urine [13,14]. It has been described that CKD patients have a progressive reduction in 1,25(OH)_2_D_3_ serum levels due to a lower glomerular filtration rate, limited 1α-hydroxylase activity, and lower megalin content [8,15], but an increase in CYP24A1 levels has also been reported [16]. The vitamin D deficiency in these patients is directly associated with mineral bone disorders, hyperphosphatemia and cardiovascular disease, which leads to accelerated disease progression and eventually death [17]. Moreover, vitamin D deficiency has been associated with epithelial barrier dysfunction and intestinal permeability in inflammatory bowel diseases (IBD), mostly due to the alteration of gut microbiome composition [18,19].

Because of the shortcomings of current dialysis techniques, novel or improved therapies that can actively secrete waste molecules and replace essential metabolic kidney functions are being sought intensively. One of the most promising solutions is a bioartificial kidney device (BAK), composed of PTEC cultured on hollow fiber membranes (HFM) to mimic proximal tubule physiology [20]. Since the first time BAK was introduced as a possible replacement kidney therapy, many studies have focused on the development and characterization of such devices [21,22,23,24,25], including human clinical trials [26], with the main limitations related to the choice of cells that would be safe and efficient enough, and readily available at the same time. A unique cell type created by our group is represented by urine-derived conditionally immortalized PTEC (ciPTEC) [27,28] equipped with the organic anion transporter 1 (OAT1) [29]. This transport protein is responsible for the uptake of many anionic waste products in kidney proximal tubule, as a first step in their renal excretion. We recently described the successful culture of ciPTEC-OAT1 on HFM and active transport of uremic toxins as active BAK component [30,31,32].

The present study was designed to characterize ciPTEC-OAT1 for the expression of genes responsible for vitamin D metabolism and function, as well as its activation to the most potent form, 1,25(OH)_2_D_3_. In addition, we evaluated the effect of a specific mixture of eight anionic uremic toxins (Table 1), mimicking uremic conditions of CKD and ESRD, on vitamin D activation and function. In particular, we focused on the beneficial effects of vitamin D on cell viability, oxidative stress, inflammation and epithelial monolayer barrier function of ciPTEC-OAT1 cultured on biofunctionalized polyethersulfone HFM. The ability of ciPTEC-OAT1 to produce vitamin D, exerting protective effects on cells, could greatly improve both BAK function and application as a treatment modality.

## 2. Results

### 2.1. Expression of Vitamin D Metabolism and Function-Related Genes in ciPTEC-OAT1

**Figure 1:**
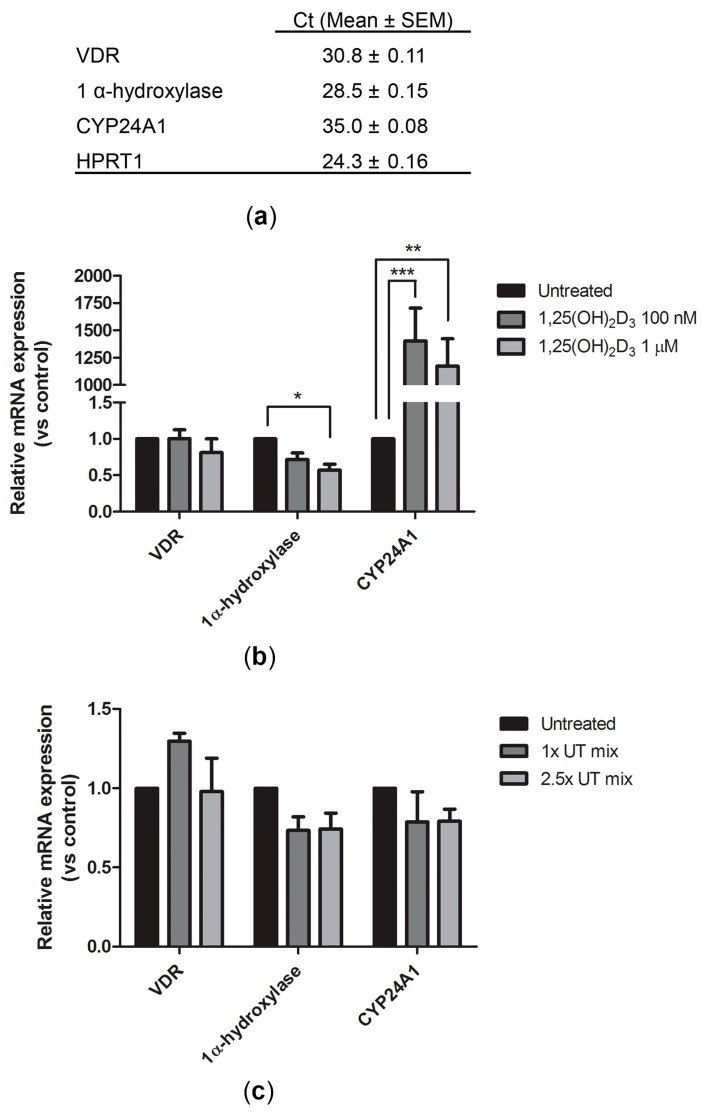
Expression of genes related to vitamin D function and metabolism in ciPTEC-OAT1. (**a**) Cycle threshold (*C*_t_) values (expressed as mean ± SEM) reflecting expression levels in basal conditions of vitamin D receptor (VDR), 1 α-hydroxylase and CYP24A1. Relative mRNA expression of three genes in ciPTEC-OAT1 after 24 h exposure to (**b**) 100 nM and 1 μM of 1,25(OH)_2_D_3_ or (**c**) 1× and 2.5× UT mix, compared to control (untreated ciPTEC-OAT1). Three independent experiments were performed in duplicate. * *p* < 0.05, ** *p* < 0.01, *** *p* < 0.001 (One-way ANOVA, Dunnett’s multiple comparison test).

In ciPTEC-OAT1, the expression of genes involved in vitamin D metabolism, activation and degradation, 1α-hydroxylase and CYP24A1, respectively, and of VDR was confirmed by real-time PCR (Figure 1a). Agarose gel electrophoresis confirmed the specificity of the primers as the size of the PCR products corresponded to the expected amplicon length (Figure S1). The housekeeping gene used for normalization was HPRT1, whose expression levels did not change upon various stimulations (Figure S2). Moreover, vitamin D’s effect on the expression of these genes was examined after 24 h exposure to either 100 nM or 1 μM of 1,25(OH)_2_D_3._ While no significant impact on VDR expression was found, the gene expression of the two enzymes was significantly affected. In fact, an almost 50% reduction in 1α-hydroxylase expression was observed after 1,25(OH)_2_D_3_ treatment when used at 1 μM, and a more than 1000-fold increase in CYP24A1 expression, regardless of the vitamin D concentration used (Figure 1b). No significant changes in gene expression were observed in the presence of a uremic toxins mixture (UT mix) at 1× or 2.5× concentrations (Figure 1c), which was used to mimic the conditions of kidney patients.

### 2.2. Conversion of 25(OH)D_3_ to 1,25(OH)_2_D_3_ by ciPTEC-OAT1

**Figure 2:**
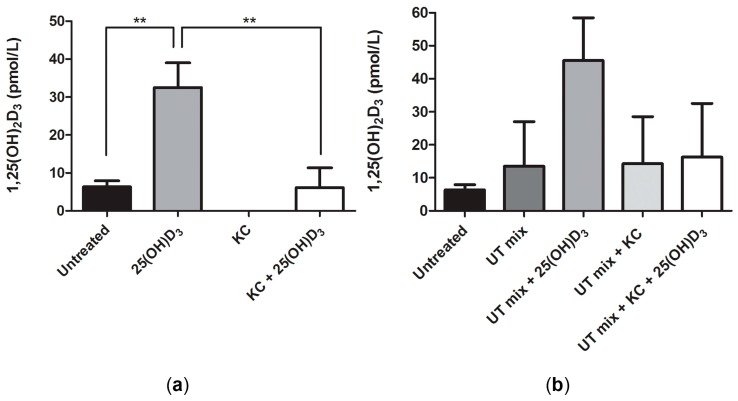
Conversion of vitamin D precursor to biologically active 1,25(OH)_2_D_3_ by ciPTEC-OAT1. (**a**) Release of 1,25(OH)_2_D_3_ in cell culture supernatant after 24 h incubation with inactive 25(OH)D_3_ (100 nM), in absence or presence of 1 α-hydroxylase inhibitor, ketoconazole (KC; 10 μM); (**b**) Release of 1,25(OH)_2_D_3_ after 24 h incubation with 25(OH)D_3_ (100 nM), in absence or presence of ketoconazole (10 μM) and 1× UT mix. Concentration expressed as pmol/L (mean ± SEM). Three independent experiments were performed. ** *p* < 0.01 (One-way ANOVA followed by Dunnett’s multiple comparison test, using as a control either untreated sample or 25(OH)D_3_ treated sample, as indicated).

To assess whether ciPTEC-OAT1 are able to produce the most active form of vitamin D, 1,25(OH)_2_D_3_, cells were exposed to 100 nM 25(OH)D_3_ for 24 h, in the presence or absence of 1α-hydroxylase inhibitor—ketoconazole (10 μM). Measured levels of 1,25(OH)_2_D_3_ confirmed that ciPTEC-OAT1 did produce the active form of vitamin D and the conversion was sensitive to inhibition by ketoconazole (Figure 2a). Uremic conditions (1× UT mix) did not influence the vitamin D activation (Figure 2b).

### 2.3. Protective Effect of 1,25(OH)_2_D_3_ on Anionic Uremic Toxin Mix Induced Cell Toxicity

**Figure 3:**
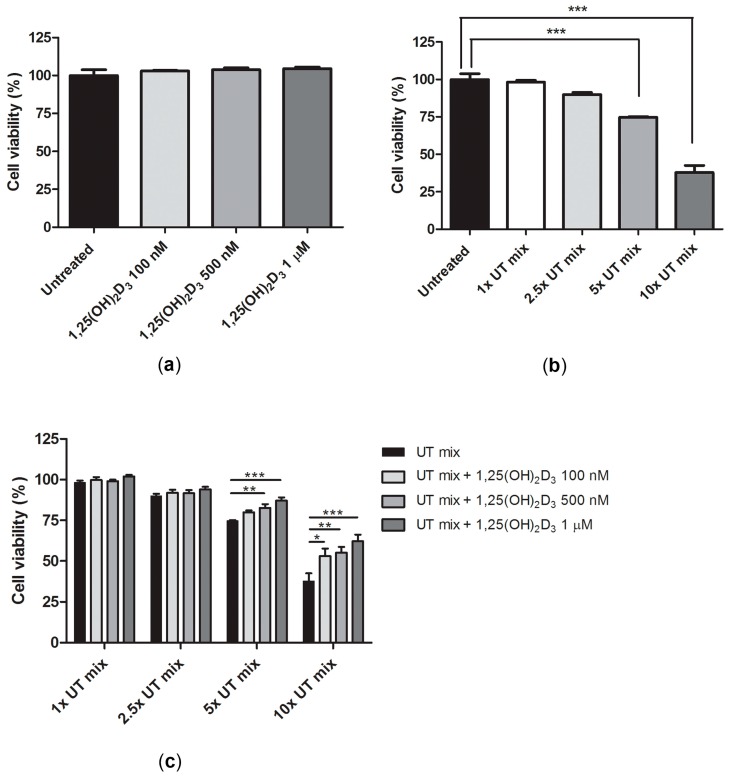
Effect of vitamin D on ciPTEC-OAT1 viability in uremic conditions. CiPTEC-OAT1 viability (relative to untreated cells) following 24 h exposure to (**a**) 1,25(OH)_2_D_3_ alone (100 nM, 500 nM and 1 μM), (**b**) increasing concentrations of UT mix (1×, 2.5×, 5×, and 10×), and (**c**) combination of 1,25(OH)_2_D_3_ and UT mix at all mentioned concentrations. Four independent experiments were performed in duplicate. * *p* < 0.05, ** *p* < 0.01, *** *p* < 0.001 (One-way ANOVA, Dunnett’s multiple comparison test).

To further examine the effect of 1,25(OH)_2_D_3_ on ciPTEC-OAT1 viability in normal and uremic conditions, cells were exposed to several concentrations of 1,25(OH)_2_D_3_ in the absence or presence of increasing concentrations of UT mix. As shown in Figure 3a, the active form of vitamin D alone did not compromise cell viability. However, anionic uremic toxins did reduce cell viability after 24 h incubation, by approximately 10%, 25%, and 62% for 2.5×, 5×, and 10× concentrated mixtures, respectively (Figure 3b). Co-incubation of 1,25(OH)_2_D_3_ with UT mix could mitigate the decrease in cell viability, especially when toxicity was induced by higher concentrations of UT mix (5× and 10×; Figure 3c).

### 2.4. Protective Effect of 1,25(OH)_2_D_3_ on Anionic Uremic Toxin Mix Induced Oxidative Stress

**Figure 4:**
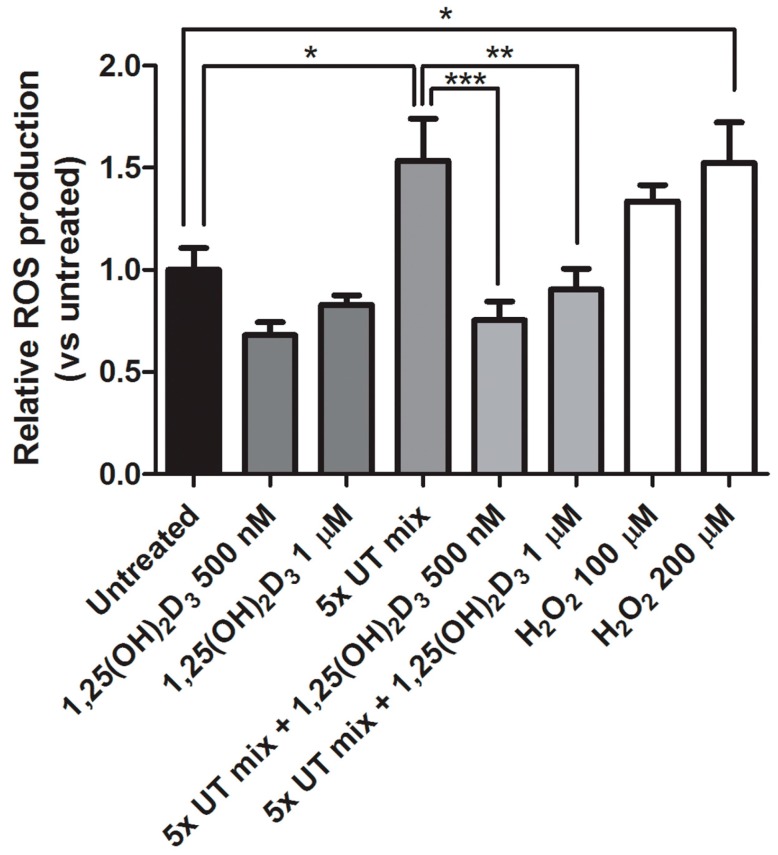
Vitamin D effect on intracellular ROS production in ciPTEC-OAT1. Relative ROS production in ciPTEC-OAT1 after 2 h exposure to 1,25(OH)_2_D_3_ (500 nM and 1 μM), 5× UT mix, combination of the two at previous concentrations and H_2_O_2_ (100 μM and 200 μM). Three independent experiments were performed in duplicate. * *p* < 0.05, ** *p* < 0.01, *** *p* < 0.001 (One-way ANOVA followed by Dunnett’s multiple comparison test, using as a control either untreated sample or 5× UT mix, as indicated).

To evaluate ciPTEC-OAT1 susceptibility to oxidative stress in uremic conditions and the anti-oxidative effect of 1,25(OH)_2_D_3_, intracellular reactive oxygen species (ROS) generation was measured. Cells were exposed to 5× UT mix, 1,25(OH)_2_D_3_ (500 nM or 1 μM) or a combination of UT mix and 1,25(OH)_2_D_3_ for 2 h. UT mix induced a 1.5-fold increase in ROS production, which was attenuated significantly when adding vitamin D as a co-treatment, regardless of concentration (Figure 4). Also, the positive control H_2_O_2_ (200 μM) significantly enhanced ROS generation (Figure 4). Using 10× UT mix, similar effects of vitamin D on intracellular ROS levels were observed (Figure S3a).

### 2.5. Anti-Inflammatory Effect of 1,25(OH)_2_D_3_ in Inflammatory and Uremic Conditions in ciPTEC-OAT1 

**Figure 5:**
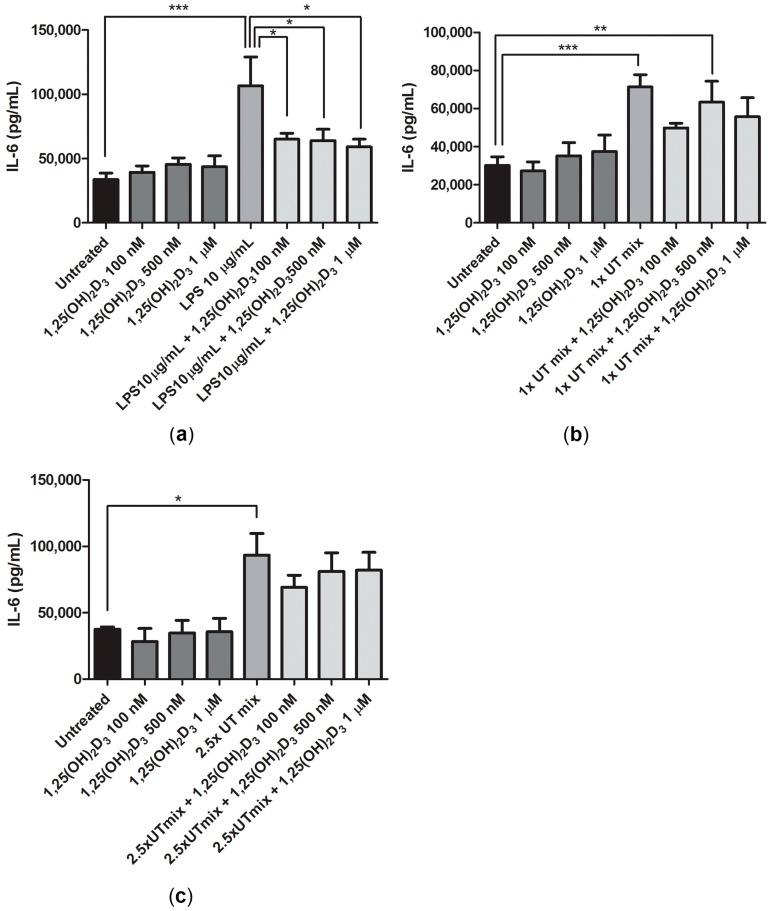
Vitamin D effect on IL-6 release by ciPTEC-OAT1 in inflammatory and uremic conditions. IL-6 release in cell culture supernatants after 24 h exposure to (**a**) 1,25(OH)_2_D_3_ (100 nM, 500 nM and 1 μM), LPS (10 μg/mL) and combination of the two, (**b**) 1,25(OH)_2_D_3_ (100 nM, 500 nM and 1 μM), 1× UT mix and combination of the two, (**c**) 1,25(OH)_2_D_3_ (100 nM, 500 nM and 1 μM), 2.5× UT mix and their combination. Concentration expressed as pg/mL (mean ± SEM). At least three independent experiments were performed. * *p* < 0.05, ** *p* < 0.01, *** *p* < 0.001 (One-way ANOVA followed by Dunnett’s multiple comparison test, using as a control either untreated sample, LPS 10 μg/mL, 1× UT mix or 2.5× UT mix, as indicated).

Interleukin-6 (IL-6) levels in cell culture supernatant were measured to assess the effect of UT mix and vitamin D on the inflammatory response of ciPTEC-OAT1. Lipopolysaccharide (LPS) (10 μg/mL), which was used as a positive control, induced a 3-fold increase in IL-6 levels after 24 h exposure. Vitamin D, however, was able to reverse this pro-inflammatory effect of LPS by reducing the IL-6 levels. A 1.6-fold reduction was found for 100 nM and 500 nM, and a 1.8-fold reduction for 1 μM of 1,25(OH)_2_D_3_ (Figure 5a). A 2-fold and 2.8-fold increase in IL-6 levels was observed following the exposure to 1× and 2.5× UT mix, respectively. Similar to what was observed for LPS, a small but evident trend in IL-6 level reduction was detected after co-treatment with 1,25(OH)_2_D_3_ (Figure 5b,c). In all conditions, TNF-α levels measured were below the limit of detection.

### 2.6. Beneficial Effect of 1,25(OH)_2_D_3_ on ciPTEC-OAT1 Epithelial Barrier Formation on HFM 

**Figure 6:**
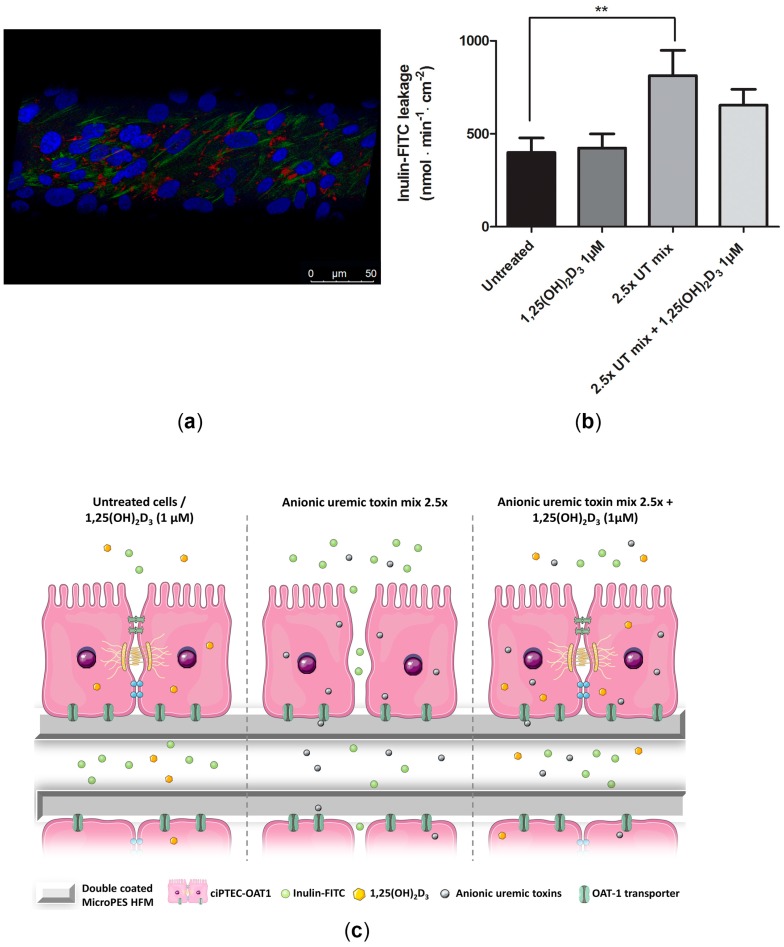
Transepithelial barrier function of ciPTEC-OAT1 cultured on hollow fiber membranes (HFM) in uremic conditions. (**a**) ciPTEC-OAT1 monolayer formation on l-DOPA (2 mg/mL) and collagen IV (25 μg/mL) coated HFM; ZO-1 expression (**red**), actin filaments (**green**) and nuclear staining with DAPI (**blue**) (25× magnification); (**b**) transepithelial inulin-FITC diffusion in fibers containing untreated cells, cells exposed to 1,25(OH)_2_D_3_ (1 μM), 2.5× UT mix and their combination for 24 h. Diffusion is expressed as nmol·min^−1^·cm^−2^ (mean ± SEM). Two independent experiments were performed in triplicate. ** *p* < 0.01 (One-way ANOVA, Dunnett’s multiple comparison test); (**c**) schematic presentation of inulin-FITC diffusion across HFM containing ciPTEC-OAT1 after various treatments.

In order to assess the stability of ciPTEC-OAT1 monolayer in uremic conditions and the effect of vitamin D on its tightness, cells were cultured on l-3,4-dihydroxyphenylalanine (l-DOPA)and collagen IV coated HFM, as described previously [30,32]. Tight monolayer was confirmed by the presence of zonula occludens 1 (ZO-1) tight junction protein and actin staining (Figure 6a). Figure 6b shows inulin-FITC diffusion through HFM containing matured monolayers of ciPTEC-OAT1, untreated, exposed to 1 μM of 1,25(OH)_2_D_3_, 2.5× UT mix or the combination of the two treatments for 24 h. UT mix increased inulin-FITC leakage to 813 ± 136 nmol·min^−1^·cm^−2^ compared to the untreated fibers (400 ± 78 nmol·min^−1^·cm^−2^; *p* < 0.01). Simultaneous exposure to 1,25(OH)_2_D_3_ could partially prevent the increase in inulin-FITC leakage induced by UT mix 2.5× (to 655 ± 85 nmol·min^−1^·cm^−2^). Figure 6c depicts a schematic presentation of inulin-FITC leakage in HFM carrying ciPTEC-OAT1 in examined conditions.

## 3. Discussion

In the present study, we demonstrated the ability of ciPTEC-OAT1 to produce the most active form of vitamin D, 1,25(OH)_2_D_3_, and its beneficial effect on various aspects of uremic conditions in ciPTEC-OAT1, including the protective effect on epithelial monolayer tightness. Considering the importance of the vitamin D deficiency often observed in CKD and ESRD, and the fact that vitamin D production is one of the main endocrine functions of proximal tubule cells, we were interested in determining whether ciPTEC-OAT1, intended for BAK purposes, possess all the necessary enzymes responsible for vitamin D metabolism. It has been shown that proximal tubule cells express 1α-hydroxylase, which is responsible for 25(OH)D_3_ conversion into 1,25(OH)_2_D_3_, as well as CYP24A1, involved in 1,25(OH)_2_D_3_ degradation [34]. Besides the proximal tubule, there are other, extra-renal sites expressing these enzymes and producing vitamin D ,such as the cells of the immune system (macrophages, monocytes, dendritic cells), and epithelial cells of the gastrointestinal tract, skin, breast, and lungs [35,36,37,38,39,40,41]. However, the major part of circulating vitamin D levels is kidney-derived, hence the severe deficiency is due to kidney failure [8]. We first evaluated the baseline expression of the genes for the activating and degrading enzymes and found that both are present in ciPTEC-OAT1, with a higher basal expression of the activating enzyme compared to the inactivating one. In line with the literature, following exposure to the active form of vitamin D, we observed a significant downregulation of 1α-hydroxylase and substantial upregulation of CYP24A1, confirming the existence of the negative feedback of 1,25(OH)_2_D_3_ on its circulating concentration [42,43]. Moreover, VDR is also expressed but not influenced by vitamin D treatment. Neither normal (1×) nor high (2.5×) concentrations of uremic toxins affected the expression of the enzymes and VDR, indicating that gene expression is not likely be altered in uremic conditions. In addition, we determined the actual conversion of 25(OH)D_3_ into 1,25(OH)_2_D_3_ in basic and uremic conditions. Cells were treated with a physiologic concentration of inactive vitamin D (100 nM, corresponding to 40 ng/mL in healthy individuals) and after 24 h the amount of active form of vitamin D generated was 32.5 pmol/L, corresponding to 13.5 pg/mL, which is slightly below the range of the active vitamin D serum levels in healthy patients [44,45]. Although speculative, this indicates that ciPTEC-OAT1 may be able to sufficiently produce the active form of vitamin D. In accordance with gene expression levels in uremic conditions, we found that the conversion of 25(OH)D_3_ was not affected by uremic toxins, suggesting a normal endocrine function of ciPTEC-OAT1 in conditions relevant to BAK applications and kidney disease.

In addition to its well-described roles, such as maintenance of calcium homeostasis and mineralization, vitamin D is able to exert other, non-calciotropic effects [46]. Among the most relevant ones are certainly immunomodulatory actions [47,48], with promotion of innate immune responses and the ability of immune system to fight infections [49,50,51], but also the suppression of the adaptive immune system with generation of tolerance, as shown for various auto-immune disorders (multiple sclerosis, type 1 diabetes, systemic lupus erythematosus and rheumatoid arthritis) [52,53,54,55]. Moreover, vitamin D is also involved in modulation of cell growth and proliferation, both in benign hyperplastic conditions and various cancer types [56].

In this study, we were particularly interested in the autocrine actions of vitamin D and therefore evaluated its effects on several cellular aspects of renal PTEC in uremic conditions. Initially, we observed that uremic toxins affect cell viability in a dose-dependent manner, while vitamin D did not have any effect. However, in the presence of anionic uremic toxins, especially at higher doses, vitamin D could restore cell viability. Numerous studies have described that some uremic toxins, such as indoxyl sulfate (IS), p-cresyl sulfate (pCS), and indole-3-acetic acid (IAA), are associated with increased inflammatory responses and oxidative stress both in vitro and in vivo [57,58,59,60,61,62,63,64,65,66]. To further address this, we measured IL-6 release by cells as an indication of inflammatory response, and ROS production, as a marker of oxidative stress, in uremic conditions and in the presence or absence of 1,25(OH)_2_D_3_ in ciPTEC-OAT1. We found that uremic toxins do increase IL-6 secreted levels, as well as ROS intracellular generation. Moreover, our results indicate that 1,25(OH)_2_D_3_ is able to reduce this increase in IL-6 levels and ROS production, confirming that vitamin D indeed has protective effects in uremic conditions, as suggested previously by in vivo studies, evaluating therapeutic effects of paricalcitol, a VDR activator, in uremic rats and hemodialysis patients [67,68,69].

A growing body of evidence suggests that vitamin D is essential for the correct functioning and maintenance of epithelial barriers, including gut mucosal barrier, corneal, pulmonary and kidney epithelial barriers, and its deficiency has been reported to promote barrier dysfunction and increased permeability [18,70,71,72]. The key tight junction proteins responsible for a tight monolayer formation in kidney proximal tubule are claudin 2 and ZO-1 [73,74]. We determined the effect of vitamin D on the stability of the proximal tubule epithelial monolayer in uremic conditions. For that purpose, ciPTEC-OAT1 were cultured on double-coated HFM to create kidney tubules consisting of mature epithelial cell monolayers, expressing both ZO-1 and claudin 2 (Figure S4a). Interestingly, we observed increased barrier permeability in the presence of uremic toxins, as shown by inulin-FITC leakage. However, in the presence of vitamin D, a clear trend towards a smaller increase in inulin-FITC diffusion was detected, suggesting the protective effect of vitamin D on the proximal tubule epithelial barrier integrity. Because the gene expression levels of ZO-1 and claudin 2 were not significantly influenced by uremic toxins or by vitamin D (Figure S4b,c), we expect that the effect of vitamin D in attenuating epithelial barrier permeability might be due to a redistribution of tight junction proteins rather than an increased protein expression, as observed previously for the intestinal barrier [18].

The findings of the present study clearly support further development of BAK as a treatment modality in patients with ESRD. Extensive previous studies described an efficient way of culturing ciPTEC-OAT1 on double-coated HFM with the formation of tight epithelial monolayers, as well as the active transport activity of both OAT1 and OCT2, proteins responsible for the clearance of uremic waste metabolites [30,32]. In addition, the lack of ciPTEC induced alloimmune response in vitro [75] and the successful upscaling of the BAK device [76] further encourage the use of these cells. Our current demonstration of the ability of the cells to activate and secrete the most active form of vitamin D is an additional important asset of the system. However, future studies should further investigate the choice of membranes used to support cell attachment, growth and monolayer formation, as this could potentially abolish the vitamin D activation function of ciPTEC-OAT1. It has been shown that some membrane types, in particular highly adsorptive and high cut-off membranes, could lead to a significant reduction in VDBP and 25(OH)D_3_ levels [6], potentially compromising the availability of 25(OH)D_3_ for megalin uptake and conversion by 1α-hydroxylase. For that reason, the polyethersulfone membranes used to support ciPTEC-OAT1 in the current settings should be tested for its suitability for use in BAK devices.

In conclusion, the ability of ciPTEC-OAT1 to produce active vitamin D could considerably boost BAK function, thus allowing the improvement of health status of kidney patients, not only by removing the excessive amounts of protein bound uremic toxins, but also by replicating one of the key endocrine functions of the proximal tubule. Eventually, the presence of 1,25(OH)_2_D_3_ would greatly contribute to the maintenance of a strong epithelial monolayers for correct and efficient BAK function, and to improved mineral homeostasis and skeletal and cardiovascular health in CKD and ESRD patients. Future experiments will be designed to evaluate the safety and efficacy of a prototype BAK device in vivo, including the assessment of the beneficial effects of vitamin D as presented in this study.

## 4. Materials and Methods

### 4.1. Reagents

All reagents (including all but two of the uremic toxins) were obtained from Sigma-Aldrich (Zwijndrecht, The Netherlands) unless stated otherwise. The uremic toxins p-cresyl sulfate (pCS) and p-cresyl glucuronide (pCG) were synthesized by the Institute for Molecules and Materials, Radboud University, Nijmegen, The Netherlands, as described [32]. Ketoconazole, 1α,25-dihydroxy-vitamin D_3_ (1,25(OH)_2_D_3_) and 25-hydroxy-vitamin D_3_ (25(OH)D_3_) were purchased form Enzo Life Sciences (Raamsdonksveer, The Netherlands). MicroPES type TF10 hollow fiber capillary membranes (wall thickness 100 μm, inner diameter 300 μm, max pore size 0.5 μm) were purchased from Membrana GmbH (Wuppertal, Germany). Cell culture plates were obtained from Greiner Bio-One (Monroe, NC, USA).

### 4.2. Cell Culture of ciPTEC-OAT1

The ciPTEC-OAT1 cell line was cultured as reported previously [29]. Briefly, cells were cultured in Dulbecco’s Modified Eagle Medium/Nutrient Mixture F-12 (1:1 DMEM/F-12) (Gibco, Life Technologies, Paisley, UK) supplemented with 10% fetal calf serum (FCS) (Greiner Bio-One, Alphen aan den Rijn, The Netherlands), 5 μg/mL insulin, 5 μg/mL transferrin, 5 μg/mL selenium, 35 ng/mL hydrocortisone, 10 ng/mL epidermal growth factor and 40 pg/mL tri-iodothyronine to form a complete culture medium, without addition of antibiotics and up to a maximum of 60 passages. Cells were cultured at 33 °C and 5% (*v*/*v*) CO_2_ to allow proliferation and prior to the experiments seeded at a density of 55,000 cell/cm^2^. Subsequently, cells were grown for one day at 33 °C, 5% (*v*/*v*) CO_2_ to allow adhesion, then cultured for seven days at 37 °C, 5% (*v*/*v*) CO_2_ for differentiation and maturation, refreshing the medium every other day.

### 4.3. ciPTEC-OAT1 Exposure to Uremic Toxins Mixture

In order to replicate the uremic conditions present in kidney patients, a specific mixture of eight known anionic uremic toxins (Table 1), predominantly derived from endogenous metabolism pathways and food digestion in the gut [33], and corresponding approximately to the concentrations found in patients (1×), or higher (2.5×, 5× and 10×) (Table 1), was used in the present study. It was prepared as a 100× concentrated mixture in a serum-free medium and subsequently diluted to desired concentrations.

### 4.4. Cell Viability Assay

Cell viability was measured using PrestoBlue^®^ cell viability reagent (Life Technologies). After seven days of maturation, cells were exposed to increasing concentrations of 1,25(OH)_2_D_3_ (100 nM, 500 nM, 1 µM), anionic UT mix (1-, 2.5-, 5-, or 10-times concentrated) and a combination of 1,25(OH)_2_D_3_ and UT mix in the previously mentioned concentrations. Following 24 h incubation at 37 °C, 5% (*v*/*v*) CO_2_, ciPTEC were rinsed once with Hank’s Balanced Salt Solution (HBSS; Gibco, Life Technologies) and incubated with PrestoBlue^®^ cell viability reagent (diluted 1:10 in complete culture medium), in the dark. After 1 h incubation at 37 °C, 5% (*v*/*v*) CO_2_, the fluorescence was measured using the Fluoroskan Ascent FL microplate reader, at excitation wavelength of 530 nm and emission wavelength of 590 nm. Data were corrected for the background, normalized to untreated cells, and presented as relative cell viability.

### 4.5. RNA Extraction, cDNA Synthesis, and Real-Time PCR

Total RNA from ciPTEC-OAT1 exposed to 1,25(OH)_2_D_3_ (100 nM and 1 µM) and UT mix (1× and 2.5×) for 24 h, was isolated using the RNeasy Mini kit (Qiagen, Venlo, The Netherlands) according to the manufacturer’s instructions and quantified using the NanoDrop^®^ ND-1000 spectrophotometer. Reverse transcription of RNA to complementary DNA (cDNA) was performed using the iScript^TM^ Reverse Transcription Supermix (Bio-Rad Laboratories, Hercules, CA, USA) following manufacturer’s instructions. Subsequently, Real-Time PCR was performed using the iQ SYBR^®^ Green Supermix (Bio-Rad Laboratories) as indicated in manufacturer’s protocol and by means of CFX96^TM^ Real-Time PCR Detection System (Bio-Rad Laboratories). The data were analyzed using Bio-Rad CFX Manager^TM^ Software version 3.1 (Bio-Rad Laboratories) and expressed as relative gene expression, using untreated cells as the reference sample. HPRT1 was used as a housekeeping gene for normalization. Specific sense and anti-sense primers for HPRT1 (forward: ACATCTGGAGTCCTATTGACATCG; reverse: CCGCCCAAAGGGAACTGATAG), VDR (forward: CTGACCCTGGAGACTTTGAC; reverse: TTCCTCTGCACTTCCTCATC), 1α-hydroxylase, (forward: GGCAGAGTCTGAATTGCAAAT; reverse: CCGGGTCTTGGGTCTAACTG), CYP24A1 (forward: GGCCTCTTTCATCACAGAGCT; reverse: GCCTATCGCGACTACCGCAA), ZO-1 (forward: ATGGTGTCCTACCTAATTCAACTCAT; reverse: GCCAGCTACAAATATTCCAACATCA) and claudin 2 (forward: ACCTGCTACCGCCACTCTGT; reverse: CTCCCTGGCCTGCATTATCTC) were synthesized by Biolegio (Nijmegen, The Netherlands).

### 4.6. Agarose Gel Electrophoresis

Real-time PCR products of the VDR, 1α-hydroxylase and CYP24A1 genes were detected by agarose gel electrophoresis. An 1.5% agarose gel was prepared in Tris-Borate-EDTA (TBE) buffer, including the SYBR^TM^ Safe DNA gel stain (1:10,000) (Invitrogen, Carlsbad, CA, USA) for visualization of cDNA fragments. Loading buffer (30% glycerol, 0.25% bromophenol blue) was added 1:6 to the PCR product samples prior to loading them in the agarose gel. The 100 bp DNA ladder (Invitrogen, Carlsbad, CA, USA) was used to determine the size of the fragments. Electrophoresis was conducted at 24 W and 100 V using Bio-Rad PowerPac^TM^ HC power supply (Bio-Rad Laboratories, Hercules, CA, USA). Following electrophoresis, the fragments were visualized using ChemiDoc^TM^ MP Imaging System (Bio-Rad Laboratories) and data analyzed by means of Image Lab software (version 5.2, Bio-Rad Laboratories).

### 4.7. Quantification of 1α,25-Dihydroxy-Vitamin D_3_

Matured ciPTEC-OAT1 were exposed to 25(OH)D_3_ 100 nM, ketoconazole 10 μM and the co-treatment, as well as 1× UT mix alone and in the presence of 25(OH)D_3_ 100 nM, ketoconazole 10 μM or both of them. Ketoconazole was always added 2 h prior to the treatment with 25(OH)D_3_ and UT mix. After 24 h incubation, cell culture supernatants were collected, centrifuged for 10 min at 240× *g*, 4 °C, and stored at −80 °C. 1,25(OH)_2_D_3_ was quantified after immuno-extraction using a competitive RIA (IDS AA-54F1; IDS Immunodiagnostic Systems GmbH, Frankfurt am Main, Germany).

### 4.8. Enzyme-Linked Immunosorbent Assay (ELISA)

The production of IL-6 and TNF-α was measured using the Enzyme-Linked Immunosorbent Assay (ELISA). Cell culture supernatants were collected after 24-h treatments with 1,25(OH)_2_D_3_ (100 nM, 500 nM, 1 µM), UT mix (1 and 2.5×), a combination of 1,25(OH)_2_D_3_ and UT mix in the previously mentioned concentrations, as well as LPS (*Escherichia coli* 0127:B8) 10 µg/mL alone or as a co-treatment with increasing concentrations of 1,25(OH)_2_D_3_. Afterwards, cell culture supernatants were centrifuged for 10 min, 240× *g*, 4 °C, and stored at −20 °C. DuoSet^®^ ELISA Development Systems kits (IL-6 #DY206, TNF-α #DY210; R&D Systems, Abingdon, UK) were used to quantify the cytokines levels in complete cell culture medium supernatants following manufacturer’s instructions. The optical density was determined using the iMark Microplate Absorbance Reader (Bio-Rad Laboratories, Hercules, CA, USA) set to 450 nm. Each sample was measured in duplicates and quantification was done using Microplate Manager software (version 6.0, Bio-Rad Laboratories), generating a four parameter logistic (4-PL) curve-fit.

### 4.9. Intracellular Reactive Oxygen Species (ROS) Detection

Intracellular ROS generation was measured by means of cell permeant fluorogenic substrate 2′,7′-dichlorofluorescein diacetate (H_2_DCFDA). Briefly, cells were washed once with HBSS, immediately loaded with H_2_DCFDA (50 μM in serum-free medium) and incubated at 37 °C, 5% (*v*/*v*) CO_2_, in the dark for 45 min. Afterwards, cells were washed with a complete culture medium and exposed to various concentrations of 1,25(OH)_2_D_3_ (500 nM and 1 µM) and 5× UT mix for 2 h at 37 °C, 5% (*v*/*v*) CO_2_, in the dark. H_2_O_2_ (100 µM and 200 µM) was used as a positive control. Following the incubation, cells were washed twice with HBSS and lysed using 0.1 M NaOH for 10 min. Finally, fluorescence was measured at an excitation wavelength of 492 nm and emission wavelength of 518 nm, using a fluorescent microplate reader (Fluoroskan Ascent FL, Thermo Fisher Scientific, Vantaa, Finland). Measured fluorescence values were corrected for the fluorescence of the blank sample (non-stained lysed cells) and used to calculate relative ROS production, using untreated cells as the reference.

### 4.10. CiPTEC-OAT1 Epithelial Monolayer Integrity

To investigate the effect of vitamin D on epithelial monolayer barrier function in uremic conditions, ciPTEC-OAT1 were cultured on l-DOPA (2 mg/mL) and collagen IV (25 µg/mL) coated HFM, mounted on a tailor-made flow chamber as described previously [30,32]. HFM with untreated mature ciPTEC-OAT1 monolayers and those exposed to 1,25(OH)_2_D_3_ (1 μM), 2.5× UT mix or a combination of both, were perfused with inulin-FITC (0.1 mg/mL) in Krebs–Henseleit buffer supplemented with 10 mM HEPES, pH 7.4, for 10 min. Next, aliquots from the apical compartment were collected and used to measure fluorescence by means of fluorescent microplate reader (Fluoroskan Ascent FL, Labsystems), at excitation wavelength of 492 nm and emission wavelength of 518 nm. Background values were subtracted and normalized arbitrary fluorescence unit (AFU) data were converted and plotted as nmol·min^−1^·cm^−2^, as described previously [32]. From each single replicate (fiber), three different regions, with an area of 0.157 cm^2^, were analyzed.

### 4.11. Immunocytochemistry

To assess the expression of tight junction protein ZO-1, ciPTEC-OAT1 cultured on double-coated HFM were fixed with 4% (*w*/*v*) paraformaldehyde dissolved in PHEM buffer (120 mM PIPES, 50 mM HEPES, 4 mM MgCl_2_, 20 mM EGTA) for 15 min. After washing the samples with HBSS, block solution (2% (*v*/*v*) FCS, 2% (*w*/*v*) bovine serum albumin (BSA), 0.1% (*v*/*v*) Tween20 in HBSS) was added. The primary antibody, rabbit anti-human ZO-1 (Invitrogen, Carlsbad, CA, USA), was diluted in blocking buffer (1:200) and incubated overnight at 4 °C. Following three washing steps with HBSS, the secondary antibody, goat anti-rabbit IgG Alexa 568 (Life Technologies, Eugene, OR, USA) was added in a concentration of 1:200 and incubated for 1 h at room temperature. Finally, ProLong^TM^ Gold antifade reagent containing DAPI (Life Technologies, Eugene, OR, USA) was used for nuclear staining, and to mount the fibers containing cells on the Willco glass bottom dishes (WillCo Wells B.V., Amsterdam, The Netherlands). Cells were imaged using confocal microscope (Leica TCS SP8 X, Leica Microsystems CMS GmbH, Wetzlar, Germany) and analyzed using Leica Application Suite X software (Leica Microsystems CMS GmbH).

### 4.12. Data Analysis

All data are presented as mean ± standard error of the mean (SEM). Statistical analysis was performed using one-way ANOVA followed by Dunnett’s multiple comparison test. A *p*-value < 0.05 was considered significant. Datasets were assessed for normality and equal variances assumptions prior to one-way ANOVA, using Kolmogorov–Smirnov and Bartlett’s tests, respectively. Even though some datasets did not meet one of the assumptions, due to a limited number of measurements, the expected effect on the Type I error in one-way ANOVA is minimal. Software used for statistical analysis was GraphPad Prism (version 6.07; GraphPad software, La Jolla, CA, USA). In most experiments at least three independent experiments were performed in duplicates, unless otherwise stated. The exact sample size for each experiment is indicated in the corresponding figure legend.

## Figures and Tables

**Table 1:** Concentrations of anionic uremic toxins in healthy individuals, uremic patients, and as applied in the present study. Concentrations used are adapted from EUToX Uremic Solutes Database (http://uremic-toxins.org/DataBase.html) and Jansen et al. [33].

Compound	Normal conc. (μM) (Mean ± SD)	Uremic conc. (μM) (Mean ± SD)	1× UT mix (μM)	Structure
Indoxyl sulfate	2.3 ± 18.8	173.5 ± 121.9	100	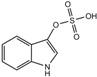
p-cresyl sulfate	10.1 ± 12.2	122.2 ± 90.3	500	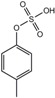
Indoxyl-β-glucuronide	3.1 ± 1.3	9.4 ± 9.4	10	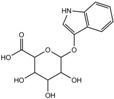
p-cresyl glucuronide	0.3 ± 0.2	30.1 ± 6.7	40	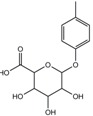
Indol-3-acetic acid	2.9 ± 1.7	11.4 ± 2.3	3	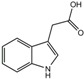
Hippuric acid	16.7 ± 11.2	608.4 ± 362.8	300	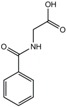
Kynurenic acid	0.03 ± 0.01	0.8 ± 0.4	3	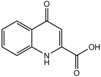
L-kynurenine	1.9	3.3 ± 0.9	5	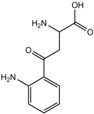

## Supplementary Materials

Supplementary materials can be found at www.mdpi.com/1422-0067/18/12/2531/s1.
