# Supplementary material for: Role of Vitamin D in Maintaining Renal Epithelial Barrier Function in Uremic Conditions
Source: Int J Mol Sci. 2017 Nov 26;18(12):2531. doi: 10.3390/ijms18122531 (PMC5751134; doi:10.3390/ijms18122531)
Supplement: Supplementary file 1 [file ijms-18-02531-s001.pdf]

Supplementary Materials: Role of Vitamin D in Maintaining Renal Epithelial Barrier Function in Uremic Conditions

Milos Mihajlovic, Michele Fedecostante, Miriam J. Oost, Sonja K. P. Steenhuis, Eef G. W. M. Lentjes, Inge Maitimu-Smeele, Manoe J. Janssen, Luuk B. Hilbrands and Rosalinde Masereeuw

Gene	Accession number	5'→3' forward primer	5'→3' reverse primer	Amplicon length
VDR	NM_000376.2	CTGACCCTGGAGACTTTGAC	TTCCTCTGCACTTCCTCATC	277
1α-hydroxylase	NM_000785.3	GGCAGAGTCTGAATTGCAAAT	CCGGGTCTTGGGTCTAACTG	97
CYP24A1	NM_000782.4	GGCCTCTTTCATCACAGAGCT	GCCTATCGCGACTACCGCAA	190

(**a**)

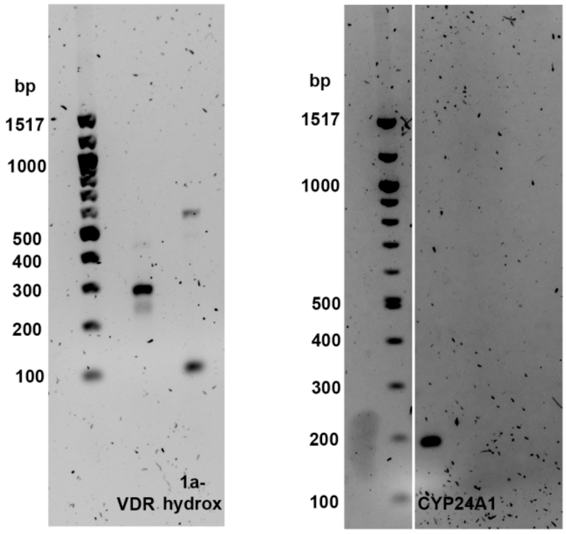

(**b**) (**c**)

Figure S1. VDR, 1 α-hydroxylase and CYP24A1 primers specificity. (**a**) Accession number, forward and reverse primer sequences and expected amplicon length of vitamin D receptor (VDR), 1α-hydroxylase and CYP24A1; (**b**,**c**) 1.5% agarose gel electrophoresis of PCR products of the three genes; all three PCR bands correspond to the expected amplicon length.

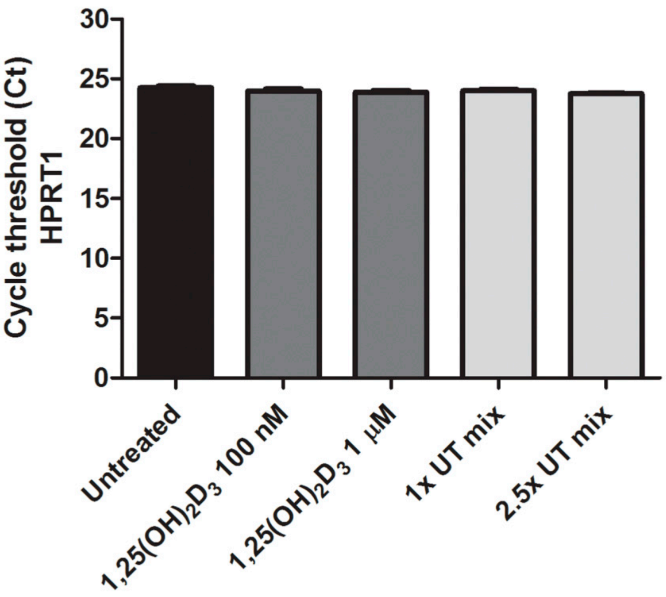

Figure S2. Cycle threshold (Ct) values for HPRT1 in different experimental conditions used in the present study. HPRT1 expression was stable, with no significant differences between various treatments. Results obtained from three independent experiments performed in duplicate. One-way ANOVA followed by Tukey multiple comparison test was used for statistical analysis.

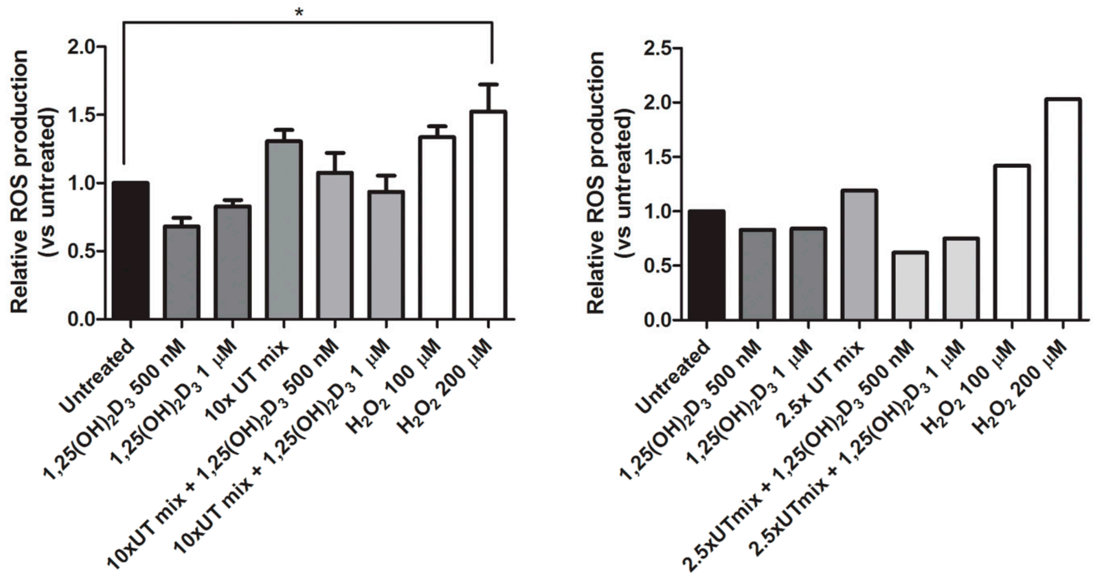

(**a**) (**b**)

Figure S3. Intracellular reactive oxygen species (ROS) production in ciPTEC-OAT1. (**a**) Relative ROS production in ciPTEC-OAT1 after 2 h exposure to 1,25(OH)$_2$D$_3$ (500 nM and 1 μM), 10x UT mix, combination of the two at previous concentrations and H_2O_2 (100 μM and 200 μM). Three independent experiments were performed in duplicate. *$p<0.05$ (One-way ANOVA, Dunnett's multiple comparison test); (**b**) Relative ROS production in ciPTEC-OAT1 after 2 h exposure to 1,25(OH)$_2$D$_3$ (500 nM and 1 μM), 2.5x UT mix, combination of the two at previous concentrations and H_2O_2 (100 μM and 200 μM).

	Ct (Mean ± SEM)
ZO-1	26.3 ± 0.16
Claudin 2	31.6 ± 0.10

(**a**)

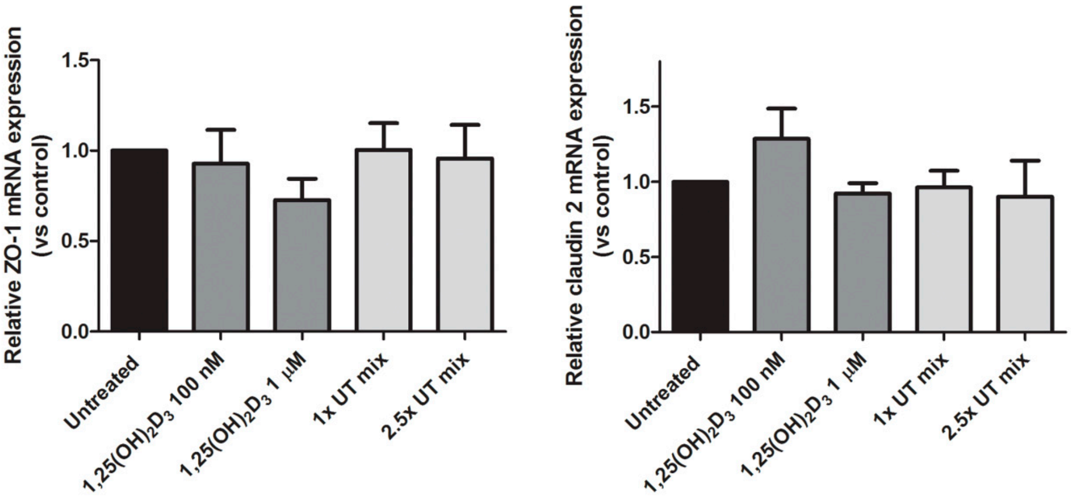

(**b**) (**c**)

Figure S4. Zonula occludens 1 (ZO-1) and claudin 2 expression in ciPTEC-OAT1. (**a**) Cycle threshold (Ct) values (expressed as mean ± SEM) reflecting ZO-1 and claudin 2 expression levels in basal conditions. Relative mRNA expression of (**b**) ZO-1 and (**c**) claudin 2 in ciPTEC-OAT1 following 24 h exposure to 100 nM and 1 μM of 1,25$(OH)_2D_3$ or 1x and 2.5x UT mix, compared to control (untreated ciPTEC-OAT1). Three independent experiments were performed in duplicate. One-way ANOVA followed by Dunnett's multiple comparison test was used for statistical analysis, and no differences were found between examined conditions.
